# Ibuprofen: The Hidden Culprit Behind Aseptic Meningitis

**DOI:** 10.7759/cureus.65936

**Published:** 2024-08-01

**Authors:** Areti Kalfoutzou, Pantelis Petroulakis, Eleni Tsiouri, Theodoros Fafoutis, Adam Mylonakis, Maria Dimitrakoudi, Maria Mylonaki, Christos Piperis, Eleni Mostratou

**Affiliations:** 1 Department of Oncology, 251 Air Force General Hospital, Athens, GRC; 2 Second Department of Internal Medicine, 251 Air Force General Hospital, Athens, GRC; 3 First Department of Surgery, Laiko General Hospital, National and Kapodistrian University of Athens, Athens, GRC; 4 Department of Hematology, Laiko General Hospital, National and Kapodistrian University of Athens, Athens, GRC; 5 Department of Radiology, Papanikolaou General Hospital, Thessaloniki, GRC; 6 Department of Cardiology, Gennimatas General Hospital, Athens, GRC

**Keywords:** delayed hypersensitivity, drugs, nsaids, aseptic meningitis, ibuprofen

## Abstract

Drug-induced aseptic meningitis represents a significant clinical entity characterized by an inflammatory response of the meninges triggered by specific pharmacological agents. This condition predominantly manifests as a delayed hypersensitivity reaction to a variety of drugs, most notably non-steroidal anti-inflammatory drugs, antibiotics, immune checkpoint inhibitors, and monoclonal antibodies. We report a case of aseptic meningitis in a 54-year-old male presenting with nausea and blurred vision two hours after taking ibuprofen. This case aims to highlight one underrecognized adverse event associated with one of the most commonly used over-the-counter medications worldwide.

## Introduction

Aseptic meningitis is a clinical syndrome characterized by the acute onset of meningeal irritation, fever, and an elevated white blood cell count in the cerebrospinal fluid (CSF) without bacterial growth in routine cultures, and may be caused by viral infections, fungal infections, parasitic infestations, certain medications, or autoimmune disorders [1]. Drug-induced aseptic meningitis (DIAM) is a rare but well-documented cause of aseptic meningitis, presenting as an adverse reaction to various medications, including non-steroidal anti-inflammatory drugs (NSAIDs), antibiotics, immune checkpoint inhibitors (ICIs), cyclooxygenase-2 (COX-2) inhibitors, and monoclonal antibodies [1,2]. The pathophysiology of DIAM is not entirely understood, but it is believed to involve a hypersensitivity reaction, given the rapid onset of symptoms following drug exposure and their resolution upon discontinuation of the offending agent. Ibuprofen, commonly used as an over-the-counter pain reliever and anti-inflammatory medication, is the drug most commonly associated with DIAM, which was first described in 1978 [3,4]. Aseptic meningitis occurs within 24 hours of ibuprofen consumption, often presenting with atypical symptoms such as headache, nausea, and blurred vision, among others. This report aims to elucidate the diagnostic challenges, clinical presentation, and management strategies for a case of ibuprofen-induced aseptic meningitis, highlighting the importance of recognizing drug-related etiologies in patients presenting with meningeal symptoms.

## Case presentation

A 54-year-old male, a school teacher by profession and a non-smoker, presented to our hospital in May 2024 with nausea and blurred vision that had started an hour earlier. His past medical history included a hospitalization for viral meningoencephalitis six months ago, and he reported no travels in the past six months, and no alcohol consumption or illicit drug use. Clinical examination revealed a febrile patient (temperature: 38.6°C). Neurological examination revealed a Glasgow Coma Scale (GCS) score of 15/15, no signs of meningeal irritation (negative Kernig and Brudzinski signs), normal muscle strength in upper and lower limbs bilaterally (5/5), and intact tendon reflexes. After a thorough medical history was obtained, the patient disclosed that he had taken 400 mg of ibuprofen approximately two hours prior to the onset of symptoms to alleviate pain following a tooth extraction. The patient was not currently on any antibiotics. He also reported experiencing a similar episode after taking ibuprofen 400 mg for a migraine one year ago.

**Figure 1:**
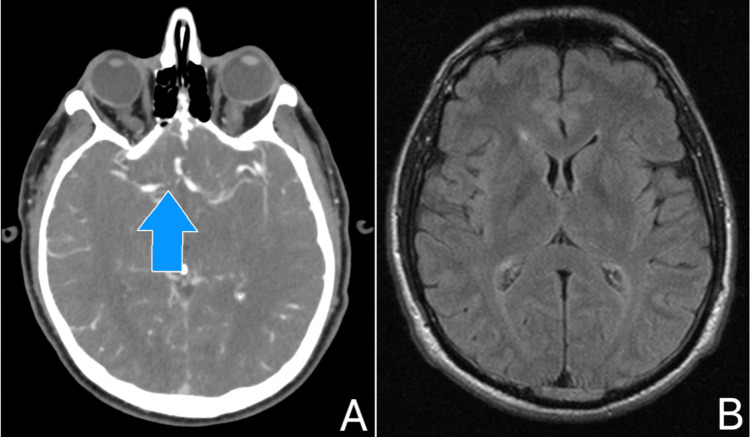
(A) Computed tomography angiography of the brain demonstrating a hypoplastic right basilar artery (blue arrow). (B) MRI scan of the brain with intravenous contrast was insignificant.

Laboratory examinations were within normal limits, except for an elevated procalcitonin level (Table 1). Blood, sputum, and urine cultures were sterile. The patient underwent a lumbar puncture (LP), and cerebrospinal fluid (CSF) analysis revealed 1600 cells/mm³, predominantly neutrophils, an elevated protein level, and a normal glucose level (Table 2). CSF cultures were sterile and viral polymerase chain reaction (PCR) assays for herpes simplex virus 1 (HSV1), herpes simplex virus 2 (HSV2), Epstein-Barr virus (EBV), cytomegalovirus (CMV), West Nile virus, adenoviruses, parvovirus B19, human herpesvirus 6 (HHV-6), human herpesvirus 8 (HHV-8), respiratory syncytial virus (RSV), and metapneumovirus in blood and CSF samples were negative. A computed tomography angiography (CTA) of the brain demonstrated a hypoplastic right basilar artery but was otherwise unremarkable (Figure 1A). Subsequently, a magnetic resonance imaging (MRI) scan of the brain with intravenous contrast showed no significant findings (Figure 1B). These findings are indicative of aseptic meningitis.

**Table 1:** Laboratory examination of the patient upon admission.

Laboratory examination	Patient’s values	Reference range
White blood cells (WBC)	9.3	4-10 K/μL
Neutrophils	6.7	1.5-7 K/μL
Hemoglobin	15.2	14-18 g/dL
Hematocrit	46.4	42-52%
Platelets	220	140-400 K/μL
Blood urea nitrogen (BUN)	52	15-54 mg/dL
Creatinine	1.2	0.7-1.3 mg/dL
Aspartate aminotransferase (AST)	12	5-40 ΙU/L
Alanine aminotransferase (ALT)	32	5-45 IU/L
C-reactive protein (CRP)	2.09	0-10 mg/L
Erythrocyte sedimentation rate (ESR)	14	0-20 mm/h
Procalcitonin	0.22	0-0.05 ng/mL
Sodium (Na)	146	137-150 mEq/L
Potassium (K)	3.8	3.5-5.3 mEq/L
Calcium (Ca)	8.2	8.1-10.4 mg/dL

**Table 2:** CSF examination of the patient during hospitalization.

Laboratory examination	Patient’s values (Day 1)	Patient’s values (Day 7)	Reference range
Cells	1600	12	0-6 cells/mm^3^
Neutrophils	80%	5%	0-6%
Protein	369	61	15-45 mg/dL
Glucose	68	64	40-70 mg/dL

The patient was initiated on empirical antibiotic treatment with intravenous ceftriaxone and metronidazole, which was subsequently escalated to ceftriaxone, vancomycin, ampicillin, and acyclovir due to persistent fever. During the 7th day of his hospitalization, he reported somnolence for the past 24 hours. Neurologic examination was unremarkable, with a GCS score of 15/15. At this point, a repeat LP demonstrated 12 cells/mm³ with a lymphocytic predominance (Table 2). Additionally, an electroencephalogram (EEG) was negative for epileptic activity.

Following a comprehensive diagnostic evaluation that excluded alternative causes of aseptic meningitis, we presumed ibuprofen-induced aseptic meningitis was the cause of the patient’s symptoms. Due to the reported association of DIAM with autoimmune disorders, an extensive immunological workup for systemic lupus erythematosus (SLE) and mixed connective tissue disease was performed, which yielded negative results.

His symptoms resolved in a few days, his clinical condition significantly improved, and he was discharged after 12 days of hospitalization. He was strongly advised to avoid taking ibuprofen as well as other NSAIDs, and he was scheduled for a follow-up appointment in one month.

## Discussion

DIAM is a type of aseptic meningitis manifesting as a delayed hypersensitivity reaction in response to a specific drug. Drugs that are frequently associated with DIAM include NSAIDs such as ibuprofen and diclofenac, antibiotics such as ciprofloxacin, metronidazole, trimethoprim-sulfamethoxazole, and cephalosporins, ICIs such as pembrolizumab and nivolumab, COX-2 inhibitors such as celecoxib, and monoclonal antibodies such as cetuximab [1,5]. Ibuprofen is the drug most frequently linked to DIAM among these pharmacologic agents [6].

Various mechanisms of ibuprofen toxicity on the meninges have been hypothesized, including a delayed hypersensitivity reaction or a direct meningeal irritation [7,8]. The most prevalent theory reports a type III or IV hypersensitivity reaction [4,6]. However, an alternative theory suggests the ibuprofen-mediated activation of a pre-existing autoantibody within the CNS, which leads to the activation of the complement system and triggers an inflammatory cascade in the meninges [9]. An association of DIAM with systemic diseases such as SLE, mixed connective tissue disease, and rheumatoid arthritis has been hypothesized [5,10].

DIAM usually manifests within 24-48 hours of drug intake [1]. About one in three cases have a previous history of taking the drug without noticing any adverse events, whereas, some patients report similar previous episodes after consuming the culprit drug [11]. The most common symptoms include headache, photophobia, fever, vomiting, blurred vision, and reduced consciousness [5]. Furthermore, neck stiffness with signs of meningeal irritation, a generalized rash, or flu-like symptoms such as myalgias may be apparent [3,9]. Our patient presented with fever and blurred vision two hours after taking ibuprofen. He had used this drug several times in the past and reported one similar episode 12 hours after using ibuprofen.

Laboratory examinations are often nonspecific but may suggest an inflammatory reaction, indicated by elevated levels of procalcitonin or C-reactive protein (CRP). CSF examination typically indicates aseptic meningitis, showing pleocytosis with a neutrophilic predominance in the absence of an identifiable pathogen [10,11]. This is accompanied by elevated CSF protein levels due to meningeal inflammation, which increases the permeability of the blood-brain barrier, as well as normal CSF glucose levels, as there is no pathogen to consume it. Laboratory examinations in our patient were insignificant, except for an elevated procalcitonin, and the initial CSF examination showed an elevated protein level and a normal glucose level, consistent with aseptic meningitis. However, a repeat CSF analysis on day seven revealed a reduced cell count and normalized protein levels, indicating a resolution of the inflammatory response in the meninges, which had previously increased the permeability of the blood-brain barrier. 

Ibuprofen-induced aseptic meningitis is usually diagnosed by exclusion of other types of meningitis, including bacterial, viral, fungal, autoimmune, or carcinomatous meningitis [1,8]. Differential diagnosis requires a comprehensive approach, including CSF analysis, blood and CSF cultures, viral PCR assays, and cytological examination to exclude infectious or malignant causes [2]. In our case, the culture results were sterile, and PCR testing was negative for all investigated pathogens.

Imaging studies such as CT and MRI scans of the brain are useful in excluding other brain pathologies, such as a pyogenic abscess. The history of recent ibuprofen consumption helps raise clinical suspicion, while a positive patch test can assist in identifying the culprit drug [1]. Repeat exposure to the suspected drug is considered unethical due to the potential for relapse and is not recommended for establishing a diagnosis [11]. In our case, the CTA scan of the brain revealed a hypoplastic right basilar artery, which was considered an incidental finding.

DIAM symptoms generally resolve rapidly within 24-48 hours after drug discontinuation [1]. Antibiotics with CNS penetrance are usually part of the initial treatment approach until infectious meningitis is safely excluded, particularly in the presence of fever or neck stiffness, whereas the use of corticosteroids remains controversial [4]. Patients should be strongly advised to avoid taking ibuprofen. Some studies recommend a lifelong avoidance of all NSAIDs and suggest substituting them with alternative anti-inflammatory or pain medications, such as paracetamol.

## Conclusions

Our case underscores the critical importance of considering DIAM in patients presenting with meningitis symptoms and a history of recent ibuprofen use. Notably, ibuprofen, while commonly used as an over-the-counter analgesic and anti-inflammatory drug, can occasionally lead to severe adverse reactions, such as aseptic meningitis, in susceptible individuals. A thorough drug history can establish the temporal relationship between drug ingestion and the emergence of symptoms. Additionally, an extensive diagnostic evaluation, including CSF analysis, blood and CSF cultures, as well as imaging studies, is necessary to exclude infectious or malignant causes. Patient education on the lifetime avoidance of ibuprofen and potentially all NSAIDs is crucial to prevent future recurrence.
